# Endothelial Dysfunction: From Pathophysiology to Novel Therapeutic Approaches

**DOI:** 10.3390/biomedicines9111571

**Published:** 2021-10-29

**Authors:** Byeong Hwa Jeon

**Affiliations:** 1 Research Institute for Medical Sciences, College of Medicine, Chungnam National University, 266 Munhwa-ro, Jung-gu, Daejeon 35015, Korea; bhjeon@cnu.ac.kr; 2 Department of Physiology, College of Medicine, Chungnam National University, 266 Munhwa-ro, Jung-gu, Daejeon 35015, Korea

The vascular endothelium is an active tissue that plays a crucial role in the maintenance of vascular homeostasis. Vascular endothelial cells in adults are composed of about 10-60 trillion cells, weighing about 1 kg and having a body surface area of 1~7 m^2^ [1]. Vascular endothelial cells are found in all tissues and are active tissues that deliver nutrients and secrete various modulators. Endothelial cells also play important roles in regulating blood flow, making them a target tissue for blood circulation [2]. The chronic exposure of risk factors, such as hypertension, hypercholesterolemia, or oxidative stress, induces endothelial dysfunctions and results in a loss of endothelial integrity, smooth muscle cell proliferation, and macrophage recruitment. In 1980, Furchgott and Zawadzki [3] discovered a substance, an endothelial cell-derived relaxing factor, and identified it as nitric oxide. This discovery was a great turning point in the development of drugs and therapeutics in the field of vascular medicine. The pathophysiology of endothelial dysfunction is complex, and multi-factorial factors are involved, such as oxidative stress or chronic inflammation. The primary prevention of cardiovascular risk factors and endothelial dysfunctions, as well as the early detection or molecular imaging techniques for endothelial dysfunction, help to prevent the development of cardiovascular disorders. Novel therapeutic approaches or drug delivery systems for endothelial dysfunctions have had a promising beneficial effect at preclinical or clinical levels, by affecting the progression of atherosclerotic changes, tumor angiogenesis, and host–immune reactions near tumor environments.

This Special Issue, entitled “Endothelial Dysfunction: From Pathophysiology to Novel Therapeutic Approaches”, is focused on the pathophysiology of endothelial dysfunction, new biomarkers for endothelial dysfunction related to cardiovascular disorders or tumors, and novel therapeutic approaches for endothelial dysfunctions. This Special Issue includes 13 review articles and 6 research papers, in which several novel biomarkers or target proteins associated with endothelial dysfunction are described. New concepts, such as new biomarkers, therapeutic targets, and treatment technologies for endothelial dysfunction are, included.

In this short editorial, I would like to highlight the paper that introduced a new concept related to endothelial cell dysfunction. Apurinic/apyrimidinic endonuclease 1/redox factor-1 (APE1/Ref-1) is an essential multifunctional protein. In 2013, the secretion of APE1/Ref-1 into the cultured medium in response to hyperacetylation was first reported [4]. Lee et al. described the usefulness of APE1/Ref-1, a novel biomarker for vascular inflammation, suggesting its potential as a serologic biomarker for cardiovascular disease [5]. APE1/Ref-1 expression is upregulated in aortic endothelial cells/macrophages of atherosclerotic mice, suggesting that plasma APE1/Ref-1 levels could predict atherosclerotic inflammation [6]. The concept of “electronegative LDL” was first proposed in 1979 [7]. By using fast-protein liquid chromatography, low-density lipoproteins (LDLs) can be divided into five subfractions (L1~L5). Among the LDL subfractions, the L5 LDL showed, in a novel concept, that it can be used as a clinical biomarker in chronic vascular thrombotic disease, including cardiometabolic disorders, acute ischemic events, and autoimmune diseases [8,9]. Chu et al. summarized that electronegative low-density lipoprotein cholesterol is a promising biomarker. A reference value of L5 LDL in serum was also presented so that this guideline for the treatment strategy could be used clinically [8]. In diabetes, vascular endothelial cell damage and endothelial cell dysfunction can be induced by changes in the activity of vascular endothelial cells and perivascular macrophages [10]. In particular, the transition from M2 (anti-inflammatory function) to M1 (inflammatory function) contributes to endothelial dysfunction and insulin resistance. Takeda et al. [11] described the mechanism of action of drugs that promote various endothelial cell functions. Sodium–glucose cotransporter 2 (SGLT2) inhibitors, glucagon-like peptide-1 (GLP-1), and dipeptidyl peptidase-4 (DDP-4) inhibitors, which inhibit M1 transition or promote the M2 macrophage, may provide good strategies to suppress endothelial dysfunction and promote the browning of white adipose tissue. 

Nannelli G et al. focused on the role of the detoxifying enzyme aldehyde dehydrogenase 2 (ALDH2) in the maintenance of endothelial function [12]. ALDH2 in mitochondria is primarily involved in the detoxification of acetaldehyde. The impairment of ALDH2 is associated with oxidative stress, aging, and endothelial dysfunction [12]. The development of therapeutic target drugs that increase the expression of ALDH2 will contribute to the development of therapeutic agents for cardiovascular diseases. In diabetes, the diverse role of glycation products needs to be investigated. Hemoglobin A1c (HbA1c) is being used as a blood biomarker, showing the chronic status of diabetes. Toma et al. summarized the role of glycated lipoprotein on endothelial cell dysfunction in diabetes and its interaction with receptors for advanced glycation end products [13]. In diabetes mellitus, the appearance of advanced glycation end products (AGE) in plasma proteins is an important etiology of endothelial dysfunction. Concepts for the glycosylation of lipoprotein, including glycated LDL or glycated HDL, would be contributed to endothelial dysfunction and/or atherosclerosis [13].

There is a new technique for treating endothelial cell dysfunction. Red and near-infrared photobiomodulation is a technology that uses light of various wavelengths to inhibit inflammation, angiogenesis, and promote blood vessel function. Although such long-wavelength light treatment technology requires extensive randomized clinical trials, it has been partially used in clinical practice [14]. Regular exercise contributes to the prevention and treatment of arteriosclerosis, diabetes, and hyperlipidemia. Regular exercise protects vascular endothelial cells and inhibits neointimal formation [15]. Proprotein convertase subtilisin/Kexin type 9 (PCSK9) is a target protein that induces arteriosclerosis, and PCSK9 antibody therapy has been developed and used in clinical practice [16]. As an interesting study, regular exercise significantly inhibits PCSK9 expression in rodent animal experiments [17].

Finally, scientific efforts for endothelium dysfunction will contribute to the development of therapeutic and preventive substances that inhibit endothelial damage in cardiovascular diseases. With the worldwide spread of COVID-19, many researchers are working to develop specific inhibitors to inhibit vascular endothelial dysfunction. We would like to thank the many scientists who participated in peer reviews, as well as the managing editors of this Special Issue of *Biomedicines*.
